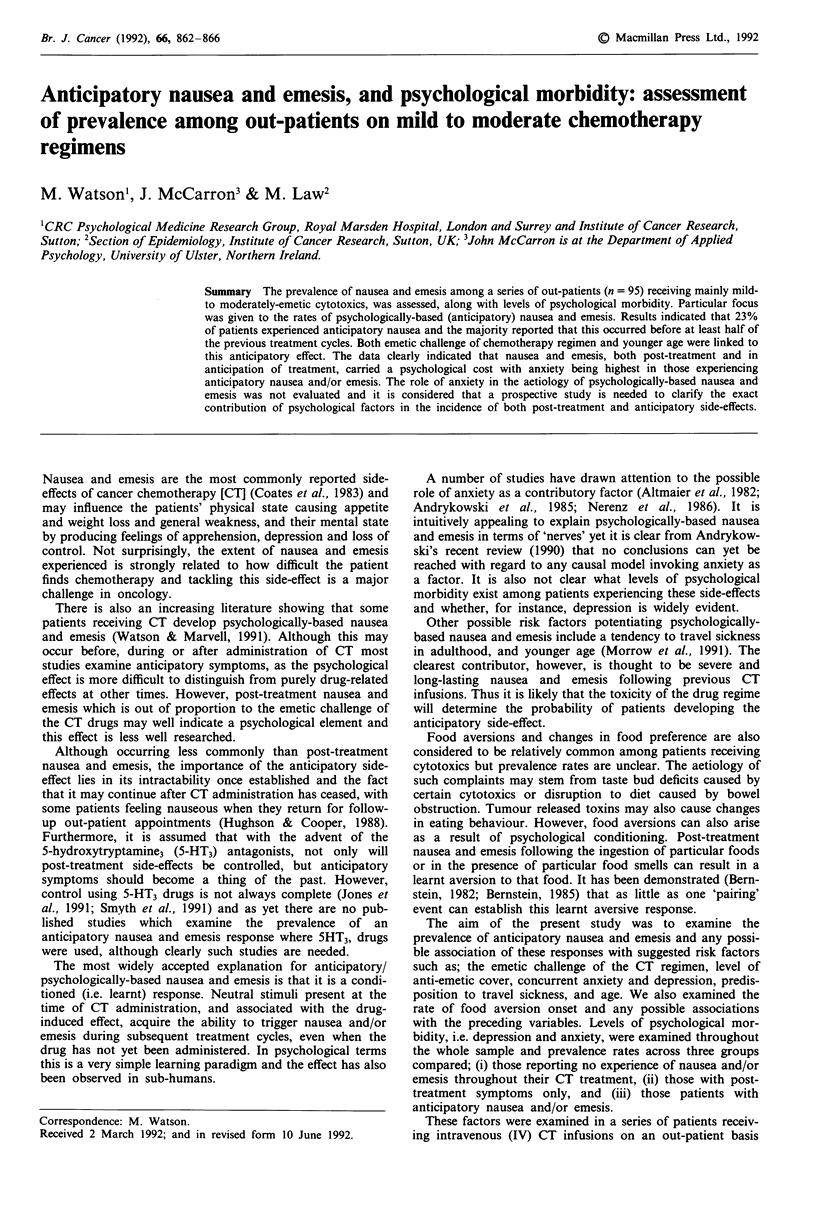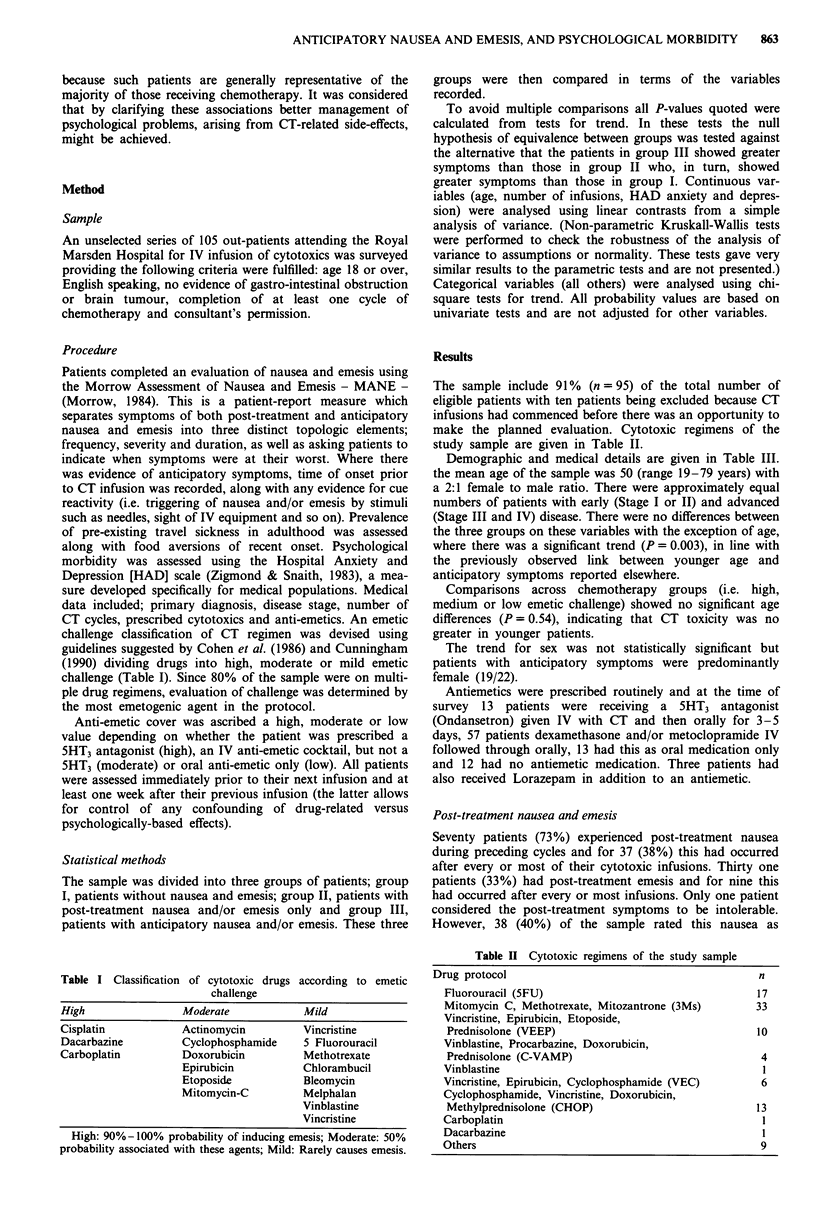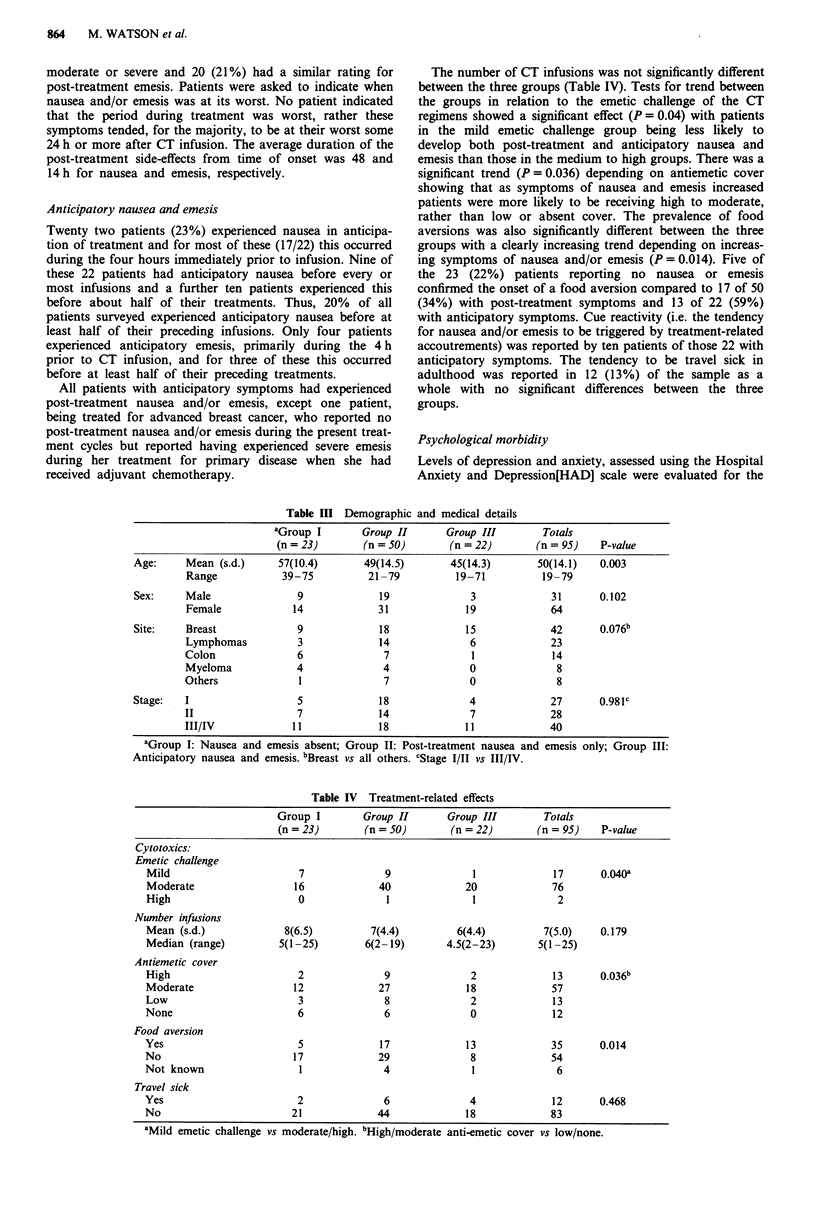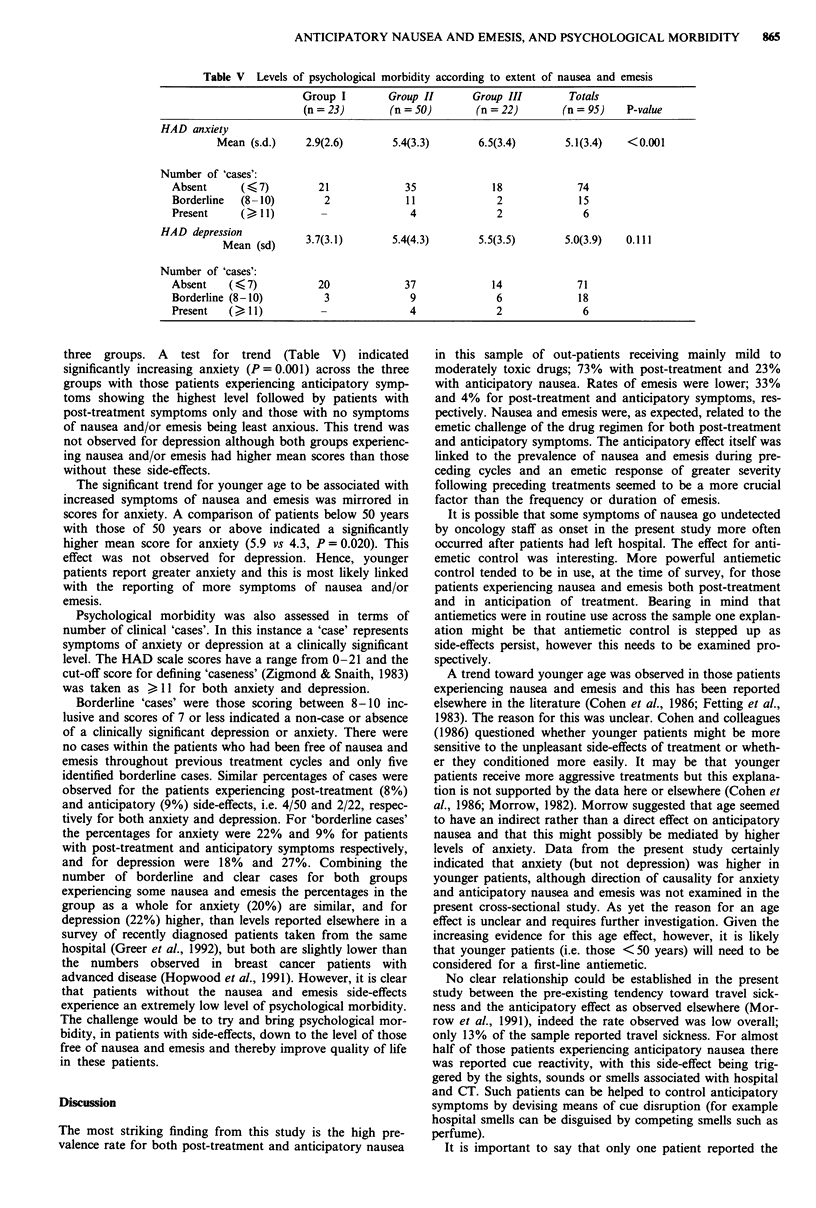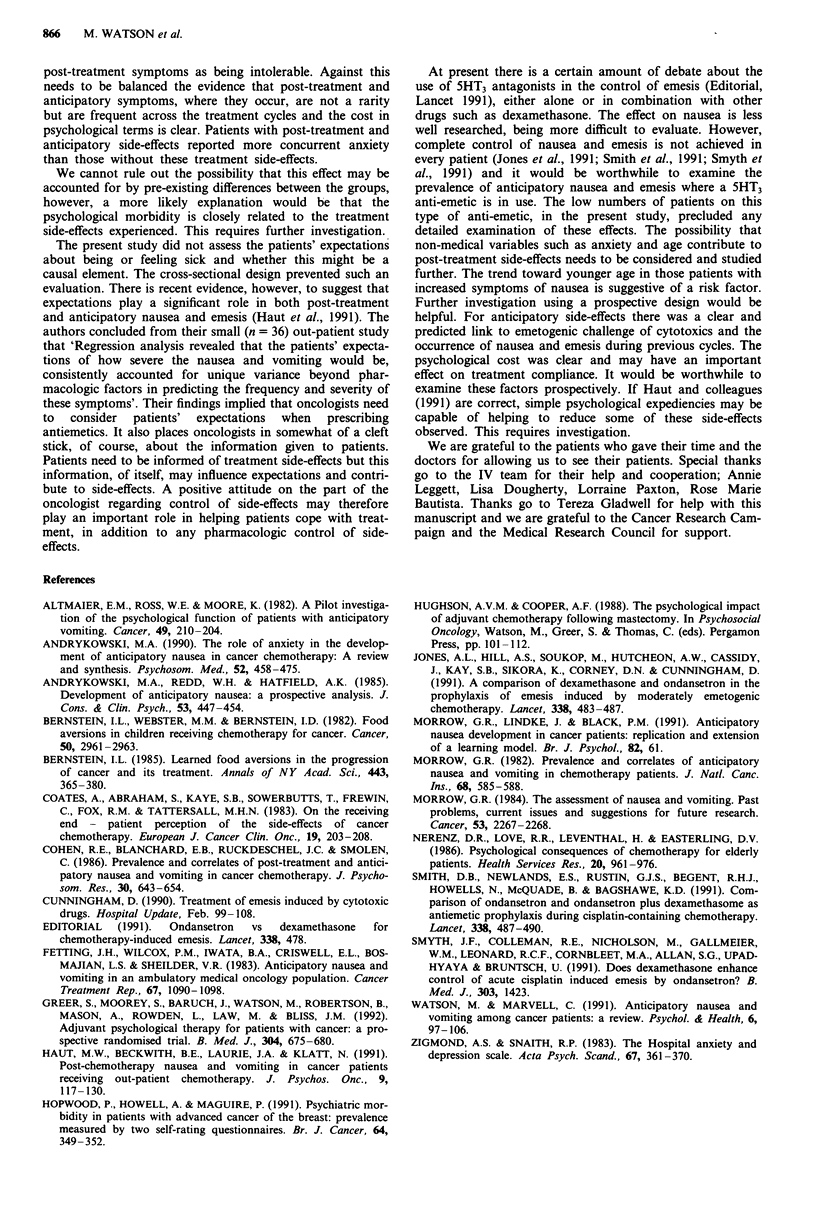# Anticipatory nausea and emesis, and psychological morbidity: assessment of prevalence among out-patients on mild to moderate chemotherapy regimens.

**DOI:** 10.1038/bjc.1992.374

**Published:** 1992-11

**Authors:** M. Watson, J. McCarron, M. Law

**Affiliations:** CRC Psychological Medicine Research Group, Royal Marsden Hospital, London, UK.

## Abstract

The prevalence of nausea and emesis among a series of out-patients (n = 95) receiving mainly mild-to moderately-emetic cytotoxics, was assessed, along with levels of psychological morbidity. Particular focus was given to the rates of psychologically-based (anticipatory) nausea and emesis. Results indicated that 23% of patients experienced anticipatory nausea and the majority reported that this occurred before at least half of the previous treatment cycles. Both emetic challenge of chemotherapy regimen and younger age were linked to this anticipatory effect. The data clearly indicated that nausea and emesis, both post-treatment and in anticipation of treatment, carried a psychological cost with anxiety being highest in those experiencing anticipatory nausea and/or emesis. The role of anxiety in the aetiology of psychologically-based nausea and emesis was not evaluated and it is considered that a prospective study is needed to clarify the exact contribution of psychological factors in the incidence of both post-treatment and anticipatory side-effects.


Br. J. Cancer (1992), 66, 862-866 © Macmillan Press Ltd., 1992

### Anticipatory nausea and emesis, and psychological morbidity: assessment of prevalence among out-patients on mild to moderate chemotherapy regimens

M. Watson[1], J. McCarron[3] & M. Law[2]

[1]*CRC Psychological Medicine Research Group, Royal Marsden Hospital, London and Surrey and Institute of Cancer Research, Sutton;* [2]*Section of Epidemiology, Institute of Cancer Research, Sutton, UK;* [3]*John McCarron is at the Department of Applied Psychology, University of Ulster, Northern Ireland.*

**Summary** The prevalence of nausea and emesis among a series of out-patients ($n = 95$) receiving mainly mild- to moderately-emetic cytotoxics, was assessed, along with levels of psychological morbidity. Particular focus was given to the rates of psychologically-based (anticipatory) nausea and emesis. Results indicated that 23% of patients experienced anticipatory nausea and the majority reported that this occurred before at least half of the previous treatment cycles. Both emetic challenge of chemotherapy regimen and younger age were linked to this anticipatory effect. The data clearly indicated that nausea and emesis, both post-treatment and in anticipation of treatment, carried a psychological cost with anxiety being highest in those experiencing anticipatory nausea and/or emesis. The role of anxiety in the aetiology of psychologically-based nausea and emesis was not evaluated and it is considered that a prospective study is needed to clarify the exact contribution of psychological factors in the incidence of both post-treatment and anticipatory side-effects.

Nausea and emesis are the most commonly reported side-effects of cancer chemotherapy [CT] (Coates *et al.*, 1983) and may influence the patients' physical state causing appetite and weight loss and general weakness, and their mental state by producing feelings of apprehension, depression and loss of control. Not surprisingly, the extent of nausea and emesis experienced is strongly related to how difficult the patient finds chemotherapy and tackling this side-effect is a major challenge in oncology.

There is also an increasing literature showing that some patients receiving CT develop psychologically-based nausea and emesis (Watson & Marvell, 1991). Although this may occur before, during or after administration of CT most studies examine anticipatory symptoms, as the psychological effect is more difficult to distinguish from purely drug-related effects at other times. However, post-treatment nausea and emesis which is out of proportion to the emetic challenge of the CT drugs may well indicate a psychological element and this effect is less well researched.

Although occurring less commonly than post-treatment nausea and emesis, the importance of the anticipatory side-effect lies in its intractability once established and the fact that it may continue after CT administration has ceased, with some patients feeling nauseous when they return for follow-up out-patient appointments (Hughson & Cooper, 1988). Furthermore, it is assumed that with the advent of the 5-hydroxytryptamine$_3$ (5-HT$_3$) antagonists, not only will post-treatment side-effects be controlled, but anticipatory symptoms should become a thing of the past. However, control using 5-HT$_3$ drugs is not always complete (Jones *et al.*, 1991; Smyth *et al.*, 1991) and as yet there are no published studies which examine the prevalence of an anticipatory nausea and emesis response where 5HT$_3$, drugs were used, although clearly such studies are needed.

The most widely accepted explanation for anticipatory/psychologically-based nausea and emesis is that it is a conditioned (i.e. learnt) response. Neutral stimuli present at the time of CT administration, and associated with the drug-induced effect, acquire the ability to trigger nausea and/or emesis during subsequent treatment cycles, even when the drug has not yet been administered. In psychological terms this is a very simple learning paradigm and the effect has also been observed in sub-humans.

Correspondence: M. Watson.
Received 2 March 1992; and in revised form 10 June 1992.

A number of studies have drawn attention to the possible role of anxiety as a contributory factor (Altmaier *et al.*, 1982; Andrykowski *et al.*, 1985; Nerenz *et al.*, 1986). It is intuitively appealing to explain psychologically-based nausea and emesis in terms of 'nerves' yet it is clear from Andrykowski's recent review (1990) that no conclusions can yet be reached with regard to any causal model invoking anxiety as a factor. It is also not clear what levels of psychological morbidity exist among patients experiencing these side-effects and whether, for instance, depression is widely evident.

Other possible risk factors potentiating psychologically-based nausea and emesis include a tendency to travel sickness in adulthood, and younger age (Morrow *et al.*, 1991). The clearest contributor, however, is thought to be severe and long-lasting nausea and emesis following previous CT infusions. Thus it is likely that the toxicity of the drug regime will determine the probability of patients developing the anticipatory side-effect.

Food aversions and changes in food preference are also considered to be relatively common among patients receiving cytotoxics but prevalence rates are unclear. The aetiology of such complaints may stem from taste bud deficits caused by certain cytotoxics or disruption to diet caused by bowel obstruction. Tumour released toxins may also cause changes in eating behaviour. However, food aversions can also arise as a result of psychological conditioning. Post-treatment nausea and emesis following the ingestion of particular foods or in the presence of particular food smells can result in a learnt aversion to that food. It has been demonstrated (Bernstein, 1982; Bernstein, 1985) that as little as one 'pairing' event can establish this learnt aversive response.

The aim of the present study was to examine the prevalence of anticipatory nausea and emesis and any possible association of these responses with suggested risk factors such as; the emetic challenge of the CT regimen, level of anti-emetic cover, concurrent anxiety and depression, predisposition to travel sickness, and age. We also examined the rate of food aversion onset and any possible associations with the preceding variables. Levels of psychological morbidity, i.e. depression and anxiety, were examined throughout the whole sample and prevalence rates across three groups compared; (i) those reporting no experience of nausea and/or emesis throughout their CT treatment, (ii) those with post-treatment symptoms only, and (iii) those patients with anticipatory nausea and/or emesis.

These factors were examined in a series of patients receiving intravenous (IV) CT infusions on an out-patient basis because such patients are generally representative of the majority of those receiving chemotherapy. It was considered that by clarifying these associations better management of psychological problems, arising from CT-related side-effects, might be achieved.

### Method

### *Sample*

An unselected series of 105 out-patients attending the Royal Marsden Hospital for IV infusion of cytotoxics was surveyed providing the following criteria were fulfilled: age 18 or over, English speaking, no evidence of gastro-intestinal obstruction or brain tumour, completion of at least one cycle of chemotherapy and consultant's permission.

### *Procedure*

Patients completed an evaluation of nausea and emesis using the Morrow Assessment of Nausea and Emesis - MANE - (Morrow, 1984). This is a patient-report measure which separates symptoms of both post-treatment and anticipatory nausea and emesis into three distinct topologic elements; frequency, severity and duration, as well as asking patients to indicate when symptoms were at their worst. Where there was evidence of anticipatory symptoms, time of onset prior to CT infusion was recorded, along with any evidence for cue reactivity (i.e. triggering of nausea and/or emesis by stimuli such as needles, sight of IV equipment and so on). Prevalence of pre-existing travel sickness in adulthood was assessed along with food aversions of recent onset. Psychological morbidity was assessed using the Hospital Anxiety and Depression [HAD] scale (Zigmond & Snaith, 1983), a measure developed specifically for medical populations. Medical data included; primary diagnosis, disease stage, number of CT cycles, prescribed cytotoxics and anti-emetics. An emetic challenge classification of CT regimen was devised using guidelines suggested by Cohen *et al.* (1986) and Cunningham (1990) dividing drugs into high, moderate or mild emetic challenge (Table I). Since 80% of the sample were on multiple drug regimens, evaluation of challenge was determined by the most emetogenic agent in the protocol.

Anti-emetic cover was ascribed a high, moderate or low value depending on whether the patient was prescribed a $5HT_3$ antagonist (high), an IV anti-emetic cocktail, but not a $5HT_3$ (moderate) or oral anti-emetic only (low). All patients were assessed immediately prior to their next infusion and at least one week after their previous infusion (the latter allows for control of any confounding of drug-related versus psychologically-based effects).

### *Statistical methods*

The sample was divided into three groups of patients; group I, patients without nausea and emesis; group II, patients with post-treatment nausea and/or emesis only and group III, patients with anticipatory nausea and/or emesis. These three groups were then compared in terms of the variables recorded.

To avoid multiple comparisons all *P*-values quoted were calculated from tests for trend. In these tests the null hypothesis of equivalence between groups was tested against the alternative that the patients in group III showed greater symptoms than those in group II who, in turn, showed greater symptoms than those in group I. Continuous variables (age, number of infusions, HAD anxiety and depression) were analysed using linear contrasts from a simple analysis of variance. (Non-parametric Kruskall-Wallis tests were performed to check the robustness of the analysis of variance to assumptions or normality. These tests gave very similar results to the parametric tests and are not presented.) Categorical variables (all others) were analysed using chi-square tests for trend. All probability values are based on univariate tests and are not adjusted for other variables.

### Results

The sample include 91% ($n = 95$) of the total number of eligible patients with ten patients being excluded because CT infusions had commenced before there was an opportunity to make the planned evaluation. Cytotoxic regimens of the study sample are given in Table II.

Demographic and medical details are given in Table III. the mean age of the sample was 50 (range 19-79 years) with a 2:1 female to male ratio. There were approximately equal numbers of patients with early (Stage I or II) and advanced (Stage III and IV) disease. There were no differences between the three groups on these variables with the exception of age, where there was a significant trend ($P = 0.003$), in line with the previously observed link between younger age and anticipatory symptoms reported elsewhere.

Comparisons across chemotherapy groups (i.e. high, medium or low emetic challenge) showed no significant age differences ($P = 0.54$), indicating that CT toxicity was no greater in younger patients.

The trend for sex was not statistically significant but patients with anticipatory symptoms were predominantly female (19/22).

Antiemetics were prescribed routinely and at the time of survey 13 patients were receiving a $5HT_3$ antagonist (Ondansetron) given IV with CT and then orally for 3-5 days, 57 patients dexamethasone and/or metoclopramide IV followed through orally, 13 had this as oral medication only and 12 had no antiemetic medication. Three patients had also received Lorazepam in addition to an antiemetic.

### *Post-treatment nausea and emesis*

Seventy patients (73%) experienced post-treatment nausea during preceding cycles and for 37 (38%) this had occurred after every or most of their cytotoxic infusions. Thirty one patients (33%) had post-treatment emesis and for nine this had occurred after every or most infusions. Only one patient considered the post-treatment symptoms to be intolerable. However, 38 (40%) of the sample rated this nausea as

**Table I** Classification of cytotoxic drugs according to emetic challenge

| *High* | *Moderate* | *Mild* |
|---|---|---|
| Cisplatin | Actinomycin | Vincristine |
| Dacarbazine | Cyclophosphamide | 5 Fluorouracil |
| Carboplatin | Doxorubicin | Methotrexate |
| | Epirubicin | Chlorambucil |
| | Etoposide | Bleomycin |
| | Mitomycin-C | Melphalan |
| | | Vinblastine |
| | | Vincristine |

High: 90%-100% probability of inducing emesis; Moderate: 50% probability associated with these agents; Mild: Rarely causes emesis.

**Table II** Cytotoxic regimens of the study sample

| Drug protocol | *n* |
|---|---|
| Fluorouracil (5FU) | 17 |
| Mitomycin C, Methotrexate, Mitozantrone (3Ms) | 33 |
| Vincristine, Epirubicin, Etoposide, Prednisolone (VEEP) | 10 |
| Vinblastine, Procarbazine, Doxorubicin, Prednisolone (C-VAMP) | 4 |
| Vinblastine | 1 |
| Vincristine, Epirubicin, Cyclophosphamide (VEC) | 6 |
| Cyclophosphamide, Vincristine, Doxorubicin, Methylprednisolone (CHOP) | 13 |
| Carboplatin | 1 |
| Dacarbazine | 1 |
| Others | 9 |

moderate or severe and 20 (21%) had a similar rating for post-treatment emesis. Patients were asked to indicate when nausea and/or emesis was at its worst. No patient indicated that the period during treatment was worst, rather these symptoms tended, for the majority, to be at their worst some 24 h or more after CT infusion. The average duration of the post-treatment side-effects from time of onset was 48 and 14 h for nausea and emesis, respectively.

*Anticipatory nausea and emesis*

Twenty two patients (23%) experienced nausea in anticipation of treatment and for most of these (17/22) this occurred during the four hours immediately prior to infusion. Nine of these 22 patients had anticipatory nausea before every or most infusions and a further ten patients experienced this before about half of their treatments. Thus, 20% of all patients surveyed experienced anticipatory nausea before at least half of their preceding infusions. Only four patients experienced anticipatory emesis, primarily during the 4 h prior to CT infusion, and for three of these this occurred before at least half of their preceding treatments.

All patients with anticipatory symptoms had experienced post-treatment nausea and/or emesis, except one patient, being treated for advanced breast cancer, who reported no post-treatment nausea and/or emesis during the present treatment cycles but reported having experienced severe emesis during her treatment for primary disease when she had received adjuvant chemotherapy.

The number of CT infusions was not significantly different between the three groups (Table IV). Tests for trend between the groups in relation to the emetic challenge of the CT regimens showed a significant effect ($P = 0.04$) with patients in the mild emetic challenge group being less likely to develop both post-treatment and anticipatory nausea and emesis than those in the medium to high groups. There was a significant trend ($P = 0.036$) depending on antiemetic cover showing that as symptoms of nausea and emesis increased patients were more likely to be receiving high to moderate, rather than low or absent cover. The prevalence of food aversions was also significantly different between the three groups with a clearly increasing trend depending on increasing symptoms of nausea and/or emesis ($P = 0.014$). Five of the 23 (22%) patients reporting no nausea or emesis confirmed the onset of a food aversion compared to 17 of 50 (34%) with post-treatment symptoms and 13 of 22 (59%) with anticipatory symptoms. Cue reactivity (i.e. the tendency for nausea and/or emesis to be triggered by treatment-related accoutrements) was reported by ten patients of those 22 with anticipatory symptoms. The tendency to be travel sick in adulthood was reported in 12 (13%) of the sample as a whole with no significant differences between the three groups.

*Psychological morbidity*

Levels of depression and anxiety, assessed using the Hospital Anxiety and Depression[HAD] scale were evaluated for the

**Table III** Demographic and medical details

| | | [a]Group I (n = 23) | Group II (n = 50) | Group III (n = 22) | Totals (n = 95) | P-value |
|---|---|---|---|---|---|---|
| Age: | Mean (s.d.) | 57(10.4) | 49(14.5) | 45(14.3) | 50(14.1) | 0.003 |
| | Range | 39–75 | 21–79 | 19–71 | 19–79 | |
| Sex: | Male | 9 | 19 | 3 | 31 | 0.102 |
| | Female | 14 | 31 | 19 | 64 | |
| Site: | Breast | 9 | 18 | 15 | 42 | 0.076[b] |
| | Lymphomas | 3 | 14 | 6 | 23 | |
| | Colon | 6 | 7 | 1 | 14 | |
| | Myeloma | 4 | 4 | 0 | 8 | |
| | Others | 1 | 7 | 0 | 8 | |
| Stage: | I | 5 | 18 | 4 | 27 | 0.981[c] |
| | II | 7 | 14 | 7 | 28 | |
| | III/IV | 11 | 18 | 11 | 40 | |

[a]Group I: Nausea and emesis absent; Group II: Post-treatment nausea and emesis only; Group III: Anticipatory nausea and emesis. [b]Breast *vs* all others. [c]Stage I/II *vs* III/IV.

**Table IV** Treatment-related effects

| | Group I (n = 23) | Group II (n = 50) | Group III (n = 22) | Totals (n = 95) | P-value |
|---|---|---|---|---|---|
| *Cytotoxics:* | | | | | |
| *Emetic challenge* | | | | | |
| Mild | 7 | 9 | 1 | 17 | 0.040[a] |
| Moderate | 16 | 40 | 20 | 76 | |
| High | 0 | 1 | 1 | 2 | |
| *Number infusions* | | | | | |
| Mean (s.d.) | 8(6.5) | 7(4.4) | 6(4.4) | 7(5.0) | 0.179 |
| Median (range) | 5(1–25) | 6(2–19) | 4.5(2–23) | 5(1–25) | |
| *Antiemetic cover* | | | | | |
| High | 2 | 9 | 2 | 13 | 0.036[b] |
| Moderate | 12 | 27 | 18 | 57 | |
| Low | 3 | 8 | 2 | 13 | |
| None | 6 | 6 | 0 | 12 | |
| *Food aversion* | | | | | |
| Yes | 5 | 17 | 13 | 35 | 0.014 |
| No | 17 | 29 | 8 | 54 | |
| Not known | 1 | 4 | 1 | 6 | |
| *Travel sick* | | | | | |
| Yes | 2 | 6 | 4 | 12 | 0.468 |
| No | 21 | 44 | 18 | 83 | |

[a]Mild emetic challenge *vs* moderate/high. [b]High/moderate anti-emetic cover *vs* low/none.

**Table V** Levels of psychological morbidity according to extent of nausea and emesis

| | Group I (n = *23)* | *Group II* (n = *50)* | *Group III* (n = *22)* | *Totals* (n = *95)* | P-*value* |
|---|---|---|---|---|---|
| *HAD anxiety* | | | | | |
| Mean (s.d.) | 2.9(2.6) | 5.4(3.3) | 6.5(3.4) | 5.1(3.4) | <0.001 |
| Number of 'cases': | | | | | |
| Absent (⩽7) | 21 | 35 | 18 | 74 | |
| Borderline (8–10) | 2 | 11 | 2 | 15 | |
| Present (⩾11) | – | 4 | 2 | 6 | |
| *HAD depression* Mean (sd) | 3.7(3.1) | 5.4(4.3) | 5.5(3.5) | 5.0(3.9) | 0.111 |
| Number of 'cases': | | | | | |
| Absent (⩽7) | 20 | 37 | 14 | 71 | |
| Borderline (8–10) | 3 | 9 | 6 | 18 | |
| Present (⩾11) | – | 4 | 2 | 6 | |

three groups. A test for trend (Table V) indicated significantly increasing anxiety ($P = 0.001$) across the three groups with those patients experiencing anticipatory symptoms showing the highest level followed by patients with post-treatment symptoms only and those with no symptoms of nausea and/or emesis being least anxious. This trend was not observed for depression although both groups experiencing nausea and/or emesis had higher mean scores than those without these side-effects.

The significant trend for younger age to be associated with increased symptoms of nausea and emesis was mirrored in scores for anxiety. A comparison of patients below 50 years with those of 50 years or above indicated a significantly higher mean score for anxiety (5.9 *vs* 4.3, $P = 0.020$). This effect was not observed for depression. Hence, younger patients report greater anxiety and this is most likely linked with the reporting of more symptoms of nausea and/or emesis.

Psychological morbidity was also assessed in terms of number of clinical 'cases'. In this instance a 'case' represents symptoms of anxiety or depression at a clinically significant level. The HAD scale scores have a range from 0–21 and the cut-off score for defining 'caseness' (Zigmond & Snaith, 1983) was taken as ⩾11 for both anxiety and depression.

Borderline 'cases' were those scoring between 8–10 inclusive and scores of 7 or less indicated a non-case or absence of a clinically significant depression or anxiety. There were no cases within the patients who had been free of nausea and emesis throughout previous treatment cycles and only five identified borderline cases. Similar percentages of cases were observed for the patients experiencing post-treatment (8%) and anticipatory (9%) side-effects, i.e. 4/50 and 2/22, respectively for both anxiety and depression. For 'borderline cases' the percentages for anxiety were 22% and 9% for patients with post-treatment and anticipatory symptoms respectively, and for depression were 18% and 27%. Combining the number of borderline and clear cases for both groups experiencing some nausea and emesis the percentages in the group as a whole for anxiety (20%) are similar, and for depression (22%) higher, than levels reported elsewhere in a survey of recently diagnosed patients taken from the same hospital (Greer *et al.*, 1992), but both are slightly lower than the numbers observed in breast cancer patients with advanced disease (Hopwood *et al.*, 1991). However, it is clear that patients without the nausea and emesis side-effects experience an extremely low level of psychological morbidity. The challenge would be to try and bring psychological morbidity, in patients with side-effects, down to the level of those free of nausea and emesis and thereby improve quality of life in these patients.

### Discussion

The most striking finding from this study is the high prevalence rate for both post-treatment and anticipatory nausea in this sample of out-patients receiving mainly mild to moderately toxic drugs; 73% with post-treatment and 23% with anticipatory nausea. Rates of emesis were lower; 33% and 4% for post-treatment and anticipatory symptoms, respectively. Nausea and emesis were, as expected, related to the emetic challenge of the drug regimen for both post-treatment and anticipatory symptoms. The anticipatory effect itself was linked to the prevalence of nausea and emesis during preceding cycles and an emetic response of greater severity following preceding treatments seemed to be a more crucial factor than the frequency or duration of emesis.

It is possible that some symptoms of nausea go undetected by oncology staff as onset in the present study more often occurred after patients had left hospital. The effect for antiemetic control was interesting. More powerful antiemetic control tended to be in use, at the time of survey, for those patients experiencing nausea and emesis both post-treatment and in anticipation of treatment. Bearing in mind that antiemetics were in routine use across the sample one explanation might be that antiemetic control is stepped up as side-effects persist, however this needs to be examined prospectively.

A trend toward younger age was observed in those patients experiencing nausea and emesis and this has been reported elsewhere in the literature (Cohen *et al.*, 1986; Fetting *et al.*, 1983). The reason for this was unclear. Cohen and colleagues (1986) questioned whether younger patients might be more sensitive to the unpleasant side-effects of treatment or whether they conditioned more easily. It may be that younger patients receive more aggressive treatments but this explanation is not supported by the data here or elsewhere (Cohen *et al.*, 1986; Morrow, 1982). Morrow suggested that age seemed to have an indirect rather than a direct effect on anticipatory nausea and that this might possibly be mediated by higher levels of anxiety. Data from the present study certainly indicated that anxiety (but not depression) was higher in younger patients, although direction of causality for anxiety and anticipatory nausea and emesis was not examined in the present cross-sectional study. As yet the reason for an age effect is unclear and requires further investigation. Given the increasing evidence for this age effect, however, it is likely that younger patients (i.e. those <50 years) will need to be considered for a first-line antiemetic.

No clear relationship could be established in the present study between the pre-existing tendency toward travel sickness and the anticipatory effect as observed elsewhere (Morrow *et al.*, 1991), indeed the rate observed was low overall; only 13% of the sample reported travel sickness. For almost half of those patients experiencing anticipatory nausea there was reported cue reactivity, with this side-effect being triggered by the sights, sounds or smells associated with hospital and CT. Such patients can be helped to control anticipatory symptoms by devising means of cue disruption (for example hospital smells can be disguised by competing smells such as perfume).

It is important to say that only one patient reported the post-treatment symptoms as being intolerable. Against this needs to be balanced the evidence that post-treatment and anticipatory symptoms, where they occur, are not a rarity but are frequent across the treatment cycles and the cost in psychological terms is clear. Patients with post-treatment and anticipatory side-effects reported more concurrent anxiety than those without these treatment side-effects.

We cannot rule out the possibility that this effect may be accounted for by pre-existing differences between the groups, however, a more likely explanation would be that the psychological morbidity is closely related to the treatment side-effects experienced. This requires further investigation.

The present study did not assess the patients' expectations about being or feeling sick and whether this might be a causal element. The cross-sectional design prevented such an evaluation. There is recent evidence, however, to suggest that expectations play a significant role in both post-treatment and anticipatory nausea and emesis (Haut et al., 1991). The authors concluded from their small ($n = 36$) out-patient study that 'Regression analysis revealed that the patients' expectations of how severe the nausea and vomiting would be, consistently accounted for unique variance beyond pharmacologic factors in predicting the frequency and severity of these symptoms'. Their findings implied that oncologists need to consider patients' expectations when prescribing antiemetics. It also places oncologists in somewhat of a cleft stick, of course, about the information given to patients. Patients need to be informed of treatment side-effects but this information, of itself, may influence expectations and contribute to side-effects. A positive attitude on the part of the oncologist regarding control of side-effects may therefore play an important role in helping patients cope with treatment, in addition to any pharmacologic control of side-effects.

At present there is a certain amount of debate about the use of $5HT_3$ antagonists in the control of emesis (Editorial, Lancet 1991), either alone or in combination with other drugs such as dexamethasone. The effect on nausea is less well researched, being more difficult to evaluate. However, complete control of nausea and emesis is not achieved in every patient (Jones et al., 1991; Smith et al., 1991; Smyth et al., 1991) and it would be worthwhile to examine the prevalence of anticipatory nausea and emesis where a $5HT_3$ anti-emetic is in use. The low numbers of patients on this type of anti-emetic, in the present study, precluded any detailed examination of these effects. The possibility that non-medical variables such as anxiety and age contribute to post-treatment side-effects needs to be considered and studied further. The trend toward younger age in those patients with increased symptoms of nausea is suggestive of a risk factor. Further investigation using a prospective design would be helpful. For anticipatory side-effects there was a clear and predicted link to emetogenic challenge of cytotoxics and the occurrence of nausea and emesis during previous cycles. The psychological cost was clear and may have an important effect on treatment compliance. It would be worthwhile to examine these factors prospectively. If Haut and colleagues (1991) are correct, simple psychological expediencies may be capable of helping to reduce some of these side-effects observed. This requires investigation.

We are grateful to the patients who gave their time and the doctors for allowing us to see their patients. Special thanks go to the IV team for their help and cooperation; Annie Leggett, Lisa Dougherty, Lorraine Paxton, Rose Marie Bautista. Thanks go to Tereza Gladwell for help with this manuscript and we are grateful to the Cancer Research Campaign and the Medical Research Council for support.

### References

ALTMAIER, E.M., ROSS, W.E. & MOORE, K. (1982). A Pilot investigation of the psychological function of patients with anticipatory vomiting. *Cancer*, **49**, 210–204.

ANDRYKOWSKI, M.A. (1990). The role of anxiety in the development of anticipatory nausea in cancer chemotherapy: A review and synthesis. *Psychosom. Med.*, **52**, 458–475.

ANDRYKOWSKI, M.A., REDD, W.H. & HATFIELD, A.K. (1985). Development of anticipatory nausea: a prospective analysis. *J. Cons. & Clin. Psych.*, **53**, 447–454.

BERNSTEIN, I.L., WEBSTER, M.M. & BERNSTEIN, I.D. (1982). Food aversions in children receiving chemotherapy for cancer. *Cancer*, **50**, 2961–2963.

BERNSTEIN, I.L. (1985). Learned food aversions in the progression of cancer and its treatment. *Annals of NY Acad. Sci.*, **443**, 365–380.

COATES, A., ABRAHAM, S., KAYE, S.B., SOWERBUTTS, T., FREWIN, C., FOX, R.M. & TATTERSALL, M.H.N. (1983). On the receiving end – patient perception of the side-effects of cancer chemotherapy. *European J. Cancer Clin. Onc.*, **19**, 203–208.

COHEN, R.E., BLANCHARD, E.B., RUCKDESCHEL, J.C. & SMOLEN, C. (1986). Prevalence and correlates of post-treatment and anticipatory nausea and vomiting in cancer chemotherapy. *J. Psychosom. Res.*, **30**, 643–654.

CUNNINGHAM, D. (1990). Treatment of emesis induced by cytotoxic drugs. *Hospital Update*, Feb. 99–108.

EDITORIAL (1991). Ondansetron vs dexamethasone for chemotherapy-induced emesis. *Lancet*, **338**, 478.

FETTING, J.H., WILCOX, P.M., IWATA, B.A., CRISWELL, E.L., BOSMAJIAN, L.S. & SHEILDER, V.R. (1983). Anticipatory nausea and vomiting in an ambulatory medical oncology population. *Cancer Treatment Rep.*, **67**, 1090–1098.

GREER, S., MOOREY, S., BARUCH, J., WATSON, M., ROBERTSON, B., MASON, A., ROWDEN, L., LAW, M. & BLISS, J.M. (1992). Adjuvant psychological therapy for patients with cancer: a prospective randomised trial. *B. Med. J.*, **304**, 675–680.

HAUT, M.W., BECKWITH, B.E., LAURIE, J.A. & KLATT, N. (1991). Post-chemotherapy nausea and vomiting in cancer patients receiving out-patient chemotherapy. *J. Psychos. Onc.*, **9**, 117–130.

HOPWOOD, P., HOWELL, A. & MAGUIRE, P. (1991). Psychiatric morbidity in patients with advanced cancer of the breast: prevalence measured by two self-rating questionnaires. *Br. J. Cancer*, **64**, 349–352.

HUGHSON, A.V.M. & COOPER, A.F. (1988). The psychological impact of adjuvant chemotherapy following mastectomy. In *Psychosocial Oncology*, Watson, M., Greer, S. & Thomas, C. (eds). Pergamon Press, pp. 101–112.

JONES, A.L., HILL, A.S., SOUKOP, M., HUTCHEON, A.W., CASSIDY, J., KAY, S.B., SIKORA, K., CORNEY, D.N. & CUNNINGHAM, D. (1991). A comparison of dexamethasone and ondansetron in the prophylaxis of emesis induced by moderately emetogenic chemotherapy. *Lancet*, **338**, 483–487.

MORROW, G.R., LINDKE, J. & BLACK, P.M. (1991). Anticipatory nausea development in cancer patients: replication and extension of a learning model. *Br. J. Psychol.*, **82**, 61.

MORROW, G.R. (1982). Prevalence and correlates of anticipatory nausea and vomiting in chemotherapy patients. *J. Natl. Canc. Ins.*, **68**, 585–588.

MORROW, G.R. (1984). The assessment of nausea and vomiting. Past problems, current issues and suggestions for future research. *Cancer*, **53**, 2267–2268.

NERENZ, D.R., LOVE, R.R., LEVENTHAL, H. & EASTERLING, D.V. (1986). Psychological consequences of chemotherapy for elderly patients. *Health Services Res.*, **20**, 961–976.

SMITH, D.B., NEWLANDS, E.S., RUSTIN, G.J.S., BEGENT, R.H.J., HOWELLS, N., McQUADE, B. & BAGSHAWE, K.D. (1991). Comparison of ondansetron and ondansetron plus dexamethasome as antiemetic prophylaxis during cisplatin-containing chemotherapy. *Lancet*, **338**, 487–490.

SMYTH, J.F., COLLEMAN, R.E., NICHOLSON, M., GALLMEIER, W.M., LEONARD, R.C.F., CORNBLEET, M.A., ALLAN, S.G., UPADHYAYA & BRUNTSCH, U. (1991). Does dexamethasone enhance control of acute cisplatin induced emesis by ondansetron? *B. Med. J.*, **303**, 1423.

WATSON, M. & MARVELL, C. (1991). Anticipatory nausea and vomiting among cancer patients: a review. *Psychol. & Health*, **6**, 97–106.

ZIGMOND, A.S. & SNAITH, R.P. (1983). The Hospital anxiety and depression scale. *Acta Psych. Scand.*, **67**, 361–370.